# Editorial: Lipid metabolism and membrane structure in plant biotic interactions

**DOI:** 10.3389/fpls.2022.1096268

**Published:** 2022-12-06

**Authors:** Fiona L. Goggin, Jyoti Shah, Glenda Gillaspy

**Affiliations:** ^1^ Department of Entomology and Plant Pathology, University of Arkansas System Division of Agriculture, Fayetteville, AR, United States; ^2^ Department of Biological Sciences and BioDiscovery Institute, University of North Texas, Denton, TX, United States; ^3^ College of Agriculture and Life Sciences, University of Wisconsin, Madison, WI, United States

**Keywords:** lipid signaling, biotic stress, sphingolipids, oxylipins, phospholipase D, inositol polyphosphates, disease resistance, pathogen resistance

## Introduction

Lipid bilayers represent the interface between cells (or organelles) and their environment. Consequently, membrane lipids and their derivatives play pivotal roles in inter- and intracellular signaling, and ultimately mediate organisms’ interactions with their biotic and abiotic environment. Plants contain an array of lipids, which include phospholipids, galactolipids, sphingolipids, and steroids. Besides their contribution as structural constituents of cellular membranes, lipids also serve as precursors for signaling metabolites that regulate plant growth, development and response to the environment (Hou et al., 2016; Lim et al., 2017). These signaling molecules include sphingolipids, lysophospholipids, and certain oxylipins such as the hormone jasmonic acid (JA), as well as secondary messengers such as phosphatidic acid (PA) and phosphoinositides (PIs) that are generated through the action of phospholipases. This special topic brings together new reports on several of these lipid classes to shed light on the impacts of plant lipid metabolism and membrane organization on plant immunity.

## Phospholipases and their products

In response to many stresses, membrane lipids are rapidly modified by lipases (Shah, 2014). Phospholipases in particular are important in generating secondary messengers such as PA and PIs that can trigger intracellular cascades such as calcium release. Besides their contribution in intracellular signaling, PIs also serve as the source of inositol polyphosphates (Ins-Ps), which are involved in phosphate homeostasis and act as messengers in intercellular signaling mediated by plant hormones. The biosynthesis of Ins-Ps in plants and their contribution to signaling is reviewed in this special issue by Riemer et al..

Phospholipases D (PLDs) are among the most common stress-responsive enzymes that modify membrane lipids. They hydrolyze phospholipids to produce PA and free head groups, promoting membrane remodeling and PA signaling. In addition, certain PLD isomers directly interact with other proteins such as G protein subunits, cytoskeletal proteins, and enzymes regulating oxidative stress signaling (Li and Wang, 2019; Deepika and Singh, 2022). In these ways PLDs and PLD-generated PA regulate a diversity of processes including cytoskeletal rearrangements, ROS generation and response, autophagy, and hormone signaling, and influence abiotic stress tolerance, pathogen resistance, and interactions with endophytes and symbionts (Camehl et al., 2011; Hong et al., 2016; Li and Wang, 2019; Zhang et al., 2021). The multiplicity of biotic and abiotic interactions that are impacted by PLDs suggest that PLDs might influence the interplay between environmental stresses and biotic interactions.

Different PLD isomers, categorized into subgroups α through ζ, vary in their catalytic properties and biological roles (Deepika and Singh, 2022). In this special topic, Yao et al. characterize the effects of soybean PLDϵ on responses to nitrogen limitation and nitrogen-fixing rhizobacteria. Compared to other PLD subgroups, relatively less is known about PLDϵ, although it is the primary isomer responding to nitrogen (N) deficiency (Hong et al., 2009; Li and Wang, 2019). Here, Yao and coworkers demonstrate that overexpression of PLDϵ in soybean can increase growth and activity of nitrogen assimilation-related enzymes under nitrogen-limited conditions. PLDϵ overexpression did not impact total nodule weight and could enhance plant growth even in the absence of rhizobia; however, it increased the accumulation of certain PA species (34:3 and 36:6 PA) in response to rhizobia, and interacted synergistically with rhizobial infection to promote seed production. Unlike overexpression of PLDα (Zhang et al., 2021), enhanced expression of PLDϵ had no negative impact on nodule formation. These results illustrate the potential applications of engineering PLD expression for improved stress resistance; moreover, they highlight the need for further research on different PLD isomers and their impact on the complex interplay between plants, their biotic interactions, and their abiotic environment.

## Sphingolipids

Sphingolipids comprise long chain bases (i.e. LCBs) and their derivatives, including ceramides (Cers), hydroxyceramides (hCers), glucosylceramides (GlcCers), and glycosylinositolphosphoceramides (GIPCs) (Quinville et al., 2021). All of these classes of sphingolipids, and particularly LCBs, have been reported to modulate plant-microbe interactions (Zeng and Yao, 2022). Synthesis of all other sphingolipids from LCBs begins through the action of ceramide synthases (CSs), which shape the profile of complex sphingolipids and also regulate LCB levels in the plants.

In this special topic, Zeng et al. compared the impacts of Class I and Class II CSs on basal plant defenses against *Pseudomonas syringae* pv. maculicola (*Psm*) in Arabidopsis. Class I CSs prefer to use a dihydroxyLCB (e.g. d18:0) and palmitoyl-CoA to form 16-Cer and other long fatty acid ceramides (LFA Cers), whereas Class II CSs prefer trihydroxyLCB (e.g. t18:0) and very-long-chain acyl-CoA as substrates to synthesize 24-Cer and other very-long-chain fatty acid Cers (Markham et al., 2011; Ternes et al., 2011). Zeng et al. results suggest that Class II CSs (LOH1 and LOH3) negatively regulate programmed cell death and other salicylic acid (SA)-dependent defenses against *Psm*, whereas a Class I CS (LOH2) may promote resistance. The *Psm*-resistant *loh1* mutant accumulated higher than normal levels of d18:0, t18:0, 16-Cer, and 24-Cer, and exogenous application of d18:0 and t18:0 induced cell death and defense gene expression in an *EDS1*-dependent manner, suggesting that the heightened levels of one or both these LCBs may contribute to bacterial resistance in *loh1* mutants. The SA signaling nodes *EDS1* and *PAD4* influenced the LCB and ceramide profiles of the *loh1* mutant, suggesting a complex interplay between sphingolipid- and SA signaling. Notably, another recent study reported that heightened t18:0 levels are likely responsible for SA- and EDS1-dependent programmed cell death in the *fah1 fah2 loh2* triple mutant, which is impaired in synthesis of possible cell death-inhibiting hCers (König et al., 2022). These results advance our understanding of the impacts of sphingolipid metabolism on plant immunity and cell death, and indicate that salicylate signaling is a key intermediary in the effect of ceramide synthases on pathogen resistance.

## Oxylipins

Oxidized lipids (oxylipins) influence programmed cell death, possess antimicrobial activities, and serve as signaling metabolites that modulate plant growth, development, and stress response (Knight et al., 2001; Hamberg et al., 2003; Wasternack and Feussner, 2018; Deboever et al., 2020). Lipoxygenases (LOXs) and dioxygenases (DOXs) contribute to the biosynthesis of oxylipins (Wasternack and Feussner, 2018). JA is one of the better studied signaling oxylipin in plants, which depending on the pathogen contributes to disease resistance or susceptibility (Yan and Xie, 2015). JA also promotes spore germination in case of *Fusarium graminearum* (Alam et al., 2022). Oxylipins are also produced by phytopathogens and influence pathogen development and virulence (Christensen and Kolomiets, 2011; Pohl and Kock, 2014). The similarities between oxylipins produced by the host and pathogen (Brodhun and Feussner, 2011; Fischer and Keller, 2016) have led to the opinion that oxylipins contribute to inter-kingdom communication between plants and phytopathogens such that some plant oxylipins facilitate pathogen development and virulence, and conversely pathogen produced oxylipins act in the host to facilitate infection (Christensen and Kolomiets, 2011; Pohl and Kock, 2014).

In this focus issue, Beccaccioli et al. review the biosynthesis and impact of fungal oxylipins on plant-fungal interaction. They further highlight recent evidence demonstrating that a similar strategy is also utilized by some bacteria to facilitate infection. They discuss recent studies with *Xylella fastidiosa*, the causative agent of olive quick decline syndrome (OQDS), that uncover the involvement of oxylipins in quorum sensing, biofilm production, motility, and virulence. They suggest that oxylipins are involved from the early stages of infection with DOX-derived oxylipins facilitating xylem colonization. Subsequently, once plant defenses have been activated, LOX-derived oxylipins accumulate causing the pathogen to switch to an ‘acquisition phase’ that promotes bacterial acquisition from xylem by the insect vector. Another study in this issue by Scala et al. utilized lipidomics with machine learning conducted on samples from OQDS-resistant and susceptible olive cultivars to show that 13-HODE, which is derived from linoleic acid (C18:2), is a biomarker for OQDS and a factor in olive trees that contributes to susceptibility to *X. fastidiosa*. The accumulation of 13-HODE correlated with increased expression of 13-LOX that putatively contribute to 13-HODE synthesis. 13-HODE had previously been shown to promote biofilm production by *X. fastidiosa* (Scala et al., 2020).

## Conclusions

Lipid-modifying enzymes such as phospholipases and lipid signals such as sphingolipids, oxylipins, PA, and InsPs play pivotal roles in inter- and intracellular signaling. Furthermore, they respond dynamically to pathogens, symbiotic microbes, insects, and other biotic agents. These dynamic changes in lipid metabolism can in some cases facilitate plant adaptation and defense, but in other cases may facilitate the colonization process by pests and pathogens. Moreover, outside factors such as environmental conditions or genetic engineering that alter lipid composition in host plants may also shift the balance between host plant resistance and susceptibility. Thus, the study of plant lipid metabolism is central to our understanding of inter-kingdom interactions.

## Author contributions

The article was conceived and written by FG, JS, and GG. All authors contributed to the article and approved the submitted version.
